# Hardness Evaluation of Composite Resins Cured with QTH and LED

**DOI:** 10.5681/joddd.2014.007

**Published:** 2014-03-05

**Authors:** Behnaz Esmaeili, Hengameh Safarcherati, Assila Vaezi

**Affiliations:** ^1^Dental Materials Research Center, Department of Operative Dentistry, School of Dentistry, Babol University of Medical Science, Babol, Iran; ^2^General Practitioner, Private Practice, Golestan Province, Iran

**Keywords:** curing lights, composite resins, dental, hardness

## Abstract

***Background and aims.*** Today light cured composites are widely used. Physical and mechanical properties of composites are related to the degree of conversion. Light curing unit (LCU) is an important factor for composite polymerization. Aim of this study is evaluation of composite resins hardness using halogen and LED light curing units.

***Materials and methods.*** In this study, 30 samples of Filtek Z250 and C-Fill composite resins were provided. Samples were light cured with Ultralume2, Valo and Astralis7. Vickers hardness number (VHN) was measured in 0, 1, 2 mm depth. Statistical analysis used: Data were analysed by SPSS software and compared with each other by T-test, one-way and two-way ANOVA and Post-hoc Tukey test.

***Results.*** In Filtek Z250, at top surface, VHN of Ultralume2 was higher than VHN of Valo (P = 0.02) and Astralis7 (P =0.04), but in depth of 1, 2 mm, VHN of Ultralume2 and Astralis7 were almost the same and both LCUs were more than Valo which the difference between Ultralume2 and Valo was significant in depth of 1mm (0.05) and 2mm (0.02). In C-Fill composite, at top surface, Astralis7 showed higher VHN, but in depth of 2 mm, performance of all devices were rather simi-lar.

***Conclusion.*** In Z250, which contains camphorquinone initiator, light cure LED Ultra-lume2 with narrow wavelength showed higher hardness number than Valo. In C-fill, in top surface, Astralis7 with more exposure time, resulted higher VHN. But In depth of 2 mm, various light curing devices had rather similar hardness number.

## Introduction

Nowadays light-cure composite resins are widely used in dentistry. The use of suitable light-emitting devices and methods are essential for polymerizing these materials. Studies have shown that the amount of composite resins’ polymerization is related to its physical-mechanical and biological characteristics.^1-5^

There are various techniques for evaluating composite polymerization which are divided into two groups: direct and indirect. Direct tests such as Fourier transform infrared (FTIR) spectroscopy are applied to determine the degree of conversion. But these techniques are rather expensive, complex and time consuming. In contrast, indirect tests such as scraping test and hardness test are inexpensive and easier to conduct.^5,9-11,13,14^Surface hardness is defined as resistance to surface indentation and it is an indirect method for measuring the degree of polymerization.

The most common and popular light curing units in dentistry are Quartz – Tungsten – Halogen devices (QTH), which were standard devices for polymerizing composites for almost two decades. However, they have some limitations such as lamp, filter and reflector part degradation, producing high temperature, short working period and extended curing time.^2,6,7^ To overcome the inherent problems of halogen light-curing units (LCUs), solid-state light-emitting diode (LED) technology has been proposed for curing light-activated dental materials. These devices have more than 10.000 hours effective lifetime. They have less degradation in continual usage, show the minimum decrease of power output and their intensity is almost constant. They are smaller and do not need filter, cooler and regular lamp replacement. Besides, their light source characteristics such as light output intensity, composition of composite material and type of photoinitiator, has influence on polymerization.

The most common photoinitiator in composite materials is camphorquinone (CQ) with spectral absorption of around 468 nm.^8,10^ However; other materials are also used as photoinitiator in composites which are activated at lower wavelengths (410-430 nm). For these materials, polymerization with LED curing units of earlier generation is questionable, due to narrower spectra of the emitted wavelengths .The light emitted from LED, has a narrower peak in its spectrum and is closer to CQ absorbing spectrum. The use of lower versions of LED devices for polymerizing composites consisting photoinitiator rather than CQ (which absorbs light at a lower wavelength than light emitted from LED) can decrease composite hardness. Uhl et al. obtained significantly lower hardness values in composites (Definite, Solitaire2) which were cured with LED curing light instead of QTH.^17^ On the other hand, Safarcherati et al. reported more hardness by LED light curing unit.^19^ In this regard it should be investigated whether LED makes desirable characteristics or not. However, the newer versions of LED have a wider output ranges and may cover the mentioned drawback.^2,7-12^, Valo light curing device has high power density (1000 mw/cm^2^) and wide spectra (395-480 nm) which may cover various photoinitiator present in different composite resins. Many studies have investigated the effect of light curing units on the hardness of composite resins, but different results have been obtained.^8,10-12,14^ The aim of this study was hardness evaluation of two composite resins cured by halogen and new LED devices. It was hypothesized that various light curing units have different effects on composite hardness.

## Materials and Methods

In this in vitro study, two kinds of microhybrid composites, Filtek Z250 and C-fill (table 1) were used. 30 samples of each composite resin (shade A_2_) were provided in brass molds which had a hole with a diameter of 8mm and a depth of 2mm. The top and bottom of the mold was covered with glass lamels during the polymerization process.

**Table 1:** Characteristics of composite resins

Composite Resin	Matrix	Filler	Manufacturer
FiltekZ250	BIS-GMA,UDMA	0.01-3.5µm	3M-ESPE
	BIS-EMA	Zr , Si	USA
C-fill	BIS-GMA	Sio2	Megadent
	2.2Bis-4(2methacryloxy-ethoxy)-phenyl-propane	Ba-Al-Br-silicate glass	Germany
	3.6Dioxaoctamethyhylen dimethacrylate		
BIS-GMA: bisphenol A glycol methacrylate			
UDMA: Urethane dimethacrylate			
BIS-EMA: ethoxylated bisphenol A glycol dimethacrylate			

In each group (n=10), composite resins were cured with light cure LED Ultralume2, halogen Astralis7 and LED Valo. In table 2, radiation time and light cure intensity of devices were mentioned. The power density of light curing devices were 400, 460 and 1000 mw/cm^2^and exposure time was adjusted to produce approximately the same energy densities (intensity × time). Before each radiation, light intensities were controlled by special LED and halogen radiometer (Demetron LED and Halogen Radiometers, Kerr, America). Light guide tip of LCUs were placed in contact with the top glass lamels during light exposure. After photo-activation the samples were kept in physiological serum in darkness and room temperature for 24 hours.

**Table 2:** Curing regimens of composite resins in terms of intensity and duration

Device	Light intensity	Radiation time	Wave length	Manufacturer
LED Valo	1000 mw/cm^2^	18.5 s	395-480 nm	Ultradent, USA
LED Ultra-lume 2	460 mw/cm^2^	40 s	440-480 nm	Ultradent, USA
QTH Astralis7	400 mw/cm^2^	46 s	400-500 nm	Ivoclar Vivadent, Austria

### Micro Hardness Evaluation 

The composite resin surfaces were polished by silicon carbide abrasive paper 400, 800, 1000, 1500, 2000, and 2500 grits consequently. Samples were cut in the central region and Vickers hardness number (VHN) was measured in 0, 1, 2 mm of depth by 500 gr load for 10s. The data were analyzed by SPSS17 software and compared with each other by T-test, one-way and two-way ANOVA and post-hoc Tukey test. The significant probability value was P value = 0.05

## Results

**Figure 1.**
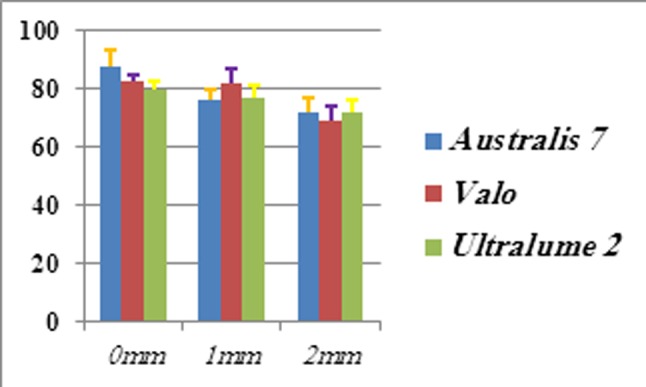
Changing trends of mean (standard deviation) micro hardness of C-fill displayed for various during devices and depths

**Figure 2.**
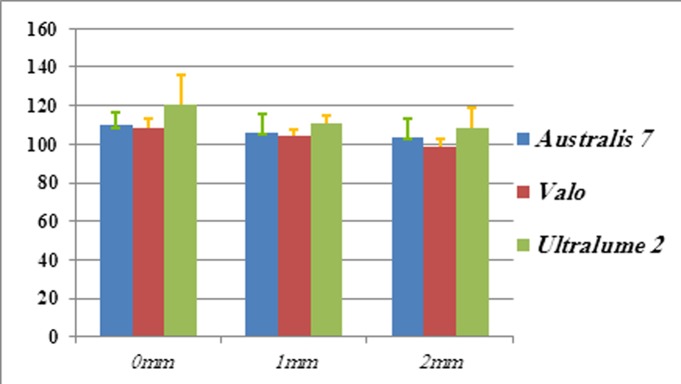
Changing trends of Mean (Standard Deviation) Micro Hardness of Filtek Z250 Displayed for Various Curing Devices and Depths

Obtained harnesses was ranged from72.21 (± 4.05) to 87.74 ( ±7.59) for C-fill and from 98.27 (± 4.04) to 120.35 ( ±14.73) for Filtek Z250. ((Figures 1,2))

The results showed that at top surface, in Filtek Z250 composite, the obtained VHN of Ultra-lume2 was significantly higher than VHN of Valo (p = 0.02) and Astralis7 (p = 0.04) ,and the two devices (Valo, Astralis7) showed no significant differences (p = 0.94). For the depth of 1 mm, VHN of Ultralume2 was higher than Astralis7 (p = 0.24) and VHN of Astralis7 was higher than Valo (p = 0.78) which had no significant differences. But the difference between Ultralume2 and Valo was significant (p = 0.05). For the depth of 2 mm the same results were obtained. The difference between Astralis7 device and Valo and Ultralume2 LED was not significant (p = 0.37) but was significant between Ultralume2 and Valo (p= 0.02).

In C-fill composite, at top surface, the obtained hardness number of Astralis7 device was significantly higher than Valo and Ultralume2 (p = 0.04 and p = 0.01, respectively) and hardness number difference between Valo and Ultralume2 was not significant (p = 0.78). For the depth of 1 mm, Valo showed higher number of hardness than Ultralume2 (p = 0.001) and Astralis7 (p = 0.002) which was statistically significant. Ultralume2 hardness number was higher than Astralis7 but it had no statistical significance (p = 0.97). For the depths of 2 mm Astralis7 showed higher number of hardness than Ultralume2 (p = 1), and Valo showed lower hardness number than Ultralume2 and Astralis7 (p = 0.3). None was preferable. In table 3 the average, standard deviation and Tukey post hoc test are shown for both composites.

## Discussion

In this in vitro study, hardness of resin composites were compared by different light curing devices. In composite Z250, at all surfaces, VHN of Ultra-lume2 was higher than the other two devices and compatible with Pris et al and safarcherati et al.^10,15^The obtained hardness number of LED device was higher than QTH. It means that the efficiency of LED unit is higher than halogen unit in Z250. Apart from light intensity and emitting time, the proper wavelength is essential for polymerization.^16^ Light energy of the three applied light devices became equal but their wavelengths were different. In Astralis7 (QTH) the wavelength was: 400–500 nm, in Ultra-lume2: 440-480 nm, and in Valo: 395–480 nm. Emitted light from Astralis7 and Valo has a broad range while light emitted from Ultralume2 is concentrated at 440-490 nm, in the most effective region for polymerization. Absorption spectrum of CQ is 440–490 nm. Z250 composite is a micro hybrid composite which contains CQ photoinitiator. As a result, Ultra-lume2 produces higher hardness number in Z250. In fact, the narrower light spectrum of Ultralume2 fits absorption peak of CQ more and better.^17^

In cases when CQ is applied as a photoinitiator, the second version of LEDs which has narrow spectrum output (450–490) and appropriate intensity (i > 300 mw/cm^2^) can make a proper polymerization in composites. But some manufacturer use less amount of CQ in composites in order to get a whiter or more translucent color in composites. They replace it with other initiator materials such as Phenyl propan dion (PPD) with absorbable spectrum of 410 nm or Bisacylphosphine oxide and Tribisacylphosphine oxide with absorbable spectrum of 320-390 nm. Gomes et al.^14^ said that combination of PPD and CQ together acts synergistically. In the first and the second versions of LEDs which have a narrow spectrum output (450–490 nm), some bondings and composites which use non-CQ photoinitiator may face with polymerization problems.^13,14,16,18,19^ As far as manufacturers do not state all photoinitiators’ combinations of their products, the efficiency of light cure devices in some composites is not predictable.

The results were different between C-fill and Filtek Z250. In C-fill QTH showed a better result on top surface and had a higher hardness number than the other devices and were similar to Polydorou et al.^1^ related to the device’s noticeable heat which led to a higher hardness number. Correr et al. showed that on the top surface of composites, exposure time is a significant factor on the polymerization.^20,21^ It seems Postirradiation conversion not to be appropriate, especially with short exposure times.^22^In this study Astralis7 had maximum exposure time (46 s) and it showed the highest VHN on top surface. It was expected that the Valo would have similar results with QTH, However, neither the higher intensity nor the wider light spectrum of the Valo resulted in a higher VHN. It probably due to the short exposure time could not have a good performance and it’s better to investigate in longer exposure time.

C-fill results were different in 1mm depth and Valo showed a higher hardness number. C-fill is a micro hybrid composite which has different components in its matrix. These components may be effective on C-fill’s different behavior to Z250. Because Valo spectrum output is wider than Ultralume2, it may cover photoinitiators with short wavelength. The Asteralis7 had the highest superficial hardness number. It appears that this surface polymerized layer due to changes in the optical properties of the composite , inhibits transmission of light through the bulk of composite despite longer exposure times.^17,20^ So Astralis7 showed lower hardness number in 1mm depth.

In the depth of 2mm of C-fill composite, no significant difference was seen between the different polymerization devices (P>0.05). In fact the devices showed rather similar functions by depth increasing and there was no significant difference between them. There is also another probability. When depth increases, light intensity decreases from refraction and light absorbance. Actually waves with longer wavelength transmit composites more and the majorities of short wavelength are absorbed near the composite surface and cannot activate other photoinitiators (co-initiator).^19,23^ Their refraction is also greater than longer wavelengths.

However, different light curing units with the same energy density don’t result necessarily the same hardness number in the composites. Moreover different shades of composite resins are available on the market than were tested in this study. Since in this study, the composites responded differently to each curing light and at different depths, so before using any brand of composites, it is better to investigate their characteristics by our light curing devices. However, more studies are needed in this field.

## Conclusion

1. In Z250 which contains camphorquinone initiator, light cure LED Ultra-lume2 with narrow wavelength showed higher hardness number than Valo.
2. In C-fill, in top surface, Astralis7 with more exposure time resulted higher VHN. But In depth of 2 mm, various light curing devices had rather similar hardness number.
3. Different LCUs with the same energy density do not result necessarily the same hardness number in the composites.

## References

[1] Polydoroun O, Manolakis A, Hellwig E, Hahn P (2008). Evaluation of the curing depth of two translucent composite materials using a halogen and two LED curing units. Clin Oral Invest.

[2] Yazici AR, Kugel G, Gul G (2007). The knoop hardness of a composite Resin polymerized with different curing lights and different modes. J Contemp Dent Pract.

[3] Tak O, Altintas SH, Ozturk N, Usumez A (2009). Effect of three types of light-curing units on 5-year color changes of light-cured composite. Clin Oral Invest.

[4] Dunn WJ, Bush AC (2002). A comparison of polymerization by light-emitting diode and halogen-based light-curing units. J Am Dent Assoc.

[5] 5-Correr AB, Sinhoreti MAC, Sobrinho LC, Tango RN, Schneider LFJ, Consani S (2005). Effect of the Increase of Energy Density on Knoop Hardness of Dental Composites Light-Cured by Conventional QTH, LED and Xenon Plasma Arc. Braz Dent J.

[6] Schneider LFJ, Consani S, Sinhoreti MAC, Crrer Sobrinho L, Milan FM (2005). Temperature change and hardness with different resin composites and phto- activation methods. Oper Dent.

[7] Auya Yazici A, Celik C, Dayangac B, Ozgunalty G (2008). Effects of different light curing unit / modes on the microleakage of flowable composite Resin. Eur J Dent.

[8] Ceballos L, Fuentes MV, Tafalla H, Martinez A, Flores J Rodriguez (2009). Curing effectiveness of resin composites at different exposure times using LED and halogen units. Med Oral Patol Oral Cir Bucal.

[9] Neo BJ, Soh M, Teo JW, Yap AU (2005). Effectiveness of composite cure associated with different light-curing regimes. Oper Dent.

[10] Peris AR, Mitsui FHO, Amaral CM, Ambrosano GMB, Pimenta LAF (2005). The effect of composite type on micro hardness when using QTH or LED light. Oper Dent.

[11] Santos GC Jr1, El-Mowafy O, Rubo JH, Santos MJ (2004). Hardening of dual-cure resin cements and a resin composite restorative cured with QTH and LED curing units. J Can Dent Assoc.

[12] Hubbezoglu I, Bolayir G, Dogan O, Ozer A, Bek B (2007). Microhardness evaluation of resin composite polymerized by three different light sources. Dent Mater.

[13] Dewald JP, Ferracane JL (1987). A comparison of four modes of evaluation depth of cure of high activated composite. J Dent Res.

[14] Gomes GM, Calixto AL, Santos FA, Gomes OMM, D’Alpino PHP, Gomes JC (2006). Hardness of a bleaching-shade resin composite polymerized with different light-curing sources. Braz Oral Res.

[15] Safarcherati H, Homayoun A (2012). Hardness of composite resin polymerized with different light-curing units. Casp J Dent Res.

[16] Prise RB, Felix CA (2009). Effect of delivering light in specific narrow bandwidths from 394 to 515 nm on the micro-hardness of resin composites. Dent Mater.

[17] Rahiotis C, Patsouri K, Silikas N, Kakaboura A (2010). Curing efficiency of high-intensity light-emitting diode devices. J Oral Science.

[18] Park SH, Kim SS, Cho YS, Lee SY, Noh BD (2005). Comparison of linear polymerization shrinkage and microhardness between QTH-cured & LED-cured composites. Oper Dent.

[19] Uhl A, Michaelis CH, Mills RW, Jandt KD (2004). The influence of storage and indentor load on the Knoop hardness of dental composites polymerized with LED and halogen technologies. Dent Mater.

[20] Correr A, Sinhoreti M, Sobrinho L, Tango R, Consani S, Schneider L (2006). Effect of exposure time vsirradiance on Knoop hardness of dental composites. Mater Res.

[21] Ceballos L, Fuentes M, Tafalla H, Martinez A, Flores J, Rodríguez J (2009). Curing effectiveness of resin composites at different exposure times using LED and halogen units. Med Oral Patol Oral Cir Bucal.

[22] Rencz A, Hickel R, Ilie N (2012). Curing efficiency of modern LED units. Clin Oral Invest.

[23] Rode KM, Freitas PM, Lloret PR, Powell LG, Turbino ML (2009). Micro-hardness evaluation of a micro-hybrid composite resin light cured with halogen light, light-emitting diode and argon ion laser. Lasers Med Sci.

